# Mr. Fawdington on Melanosis

**Published:** 1826-10-01

**Authors:** 


*A Case of Melanosis, with General Observations on the Pathology of this Interesting Disease.* By Thomas Fawdington, Member of the Royal College of Surgeons, London, and one of the Surgeons to the Manchester Lying-in-Hospital. Illustrated by Coloured Lithographic Plates. 8vo. pp. 49. Longman & Co. May, 1826.

In a very modest and unassuming preface, Mr. Fawdington informs us that the following case was originally drawn up for insertion in one of the periodical journals; but, owing to the advice of friends, and the inadequacy of mere verbal description to convey an accurate notion of morbid appearances, together with the interesting peculiarity of the disease in question, he has been induced to adopt the present mode of publication. No graphic illustrations of melanosis have been attempted, we believe, in this country, and but few on the Continent; and it is but justice to say, that the plates, in the present work, are admirably executed, and highly calculated to convey a most accurate idea of a disease with which the eye of the practitioner is but little familiar—though instances of the complaint are not exceedingly rare, as our journals have lately proved. Melanosis is sometimes passed over as a strange and anomalous appearance, not understood, and we think the graphic delineations in this book are perfectly adequate to prevent all misapprehension or mistake on such occasions in future.

Melanosis has but recently attracted the notice of the profession, though some faint traces of the disease are scattered in the works of Morgagni, Haller, and Bonetus. Bayle, Laennec, Dupuytren, and Breschet have accurately described this morbid state in France, and several cases have been published in this country—one of which, by Dr. Gregory, will be fresh in the minds of our readers. "No where," says our author, "has melanosis been observed presenting a more complete and unmixed character than in the case which occupies the following pages," and to the particulars of this case we shall now direct our attention.

*Case.* Thomas Peckett, aged 30, a robust and healthy looking man, a carder in a cotton mill, consulted Mr. Wilson, senior surgeon to the Manchester Eye Infirmary, in January, 1824, respecting a violent and incessant pain in his left eye, on which organ he had received a blow by a piece of iron six months before. A fortnight after this accident he experienced a sensation of fulness in the globe, and the sight had become affected. These symptoms gradually increased—the conjunctival vessels became enlarged, tortuous, and fasciculated—the sclerotic inflamed and attenuated—the dark choroid becoming visible—the iris dilated and immoveable—and a slate-coloured opacity occupying the centre of the pupil. The usual means were resorted to, but without any other good effect than the removal of the pain. In the latter end of March, the pain again returned, and deprived the patient of sleep. The disease had now made considerable progress, and it was feared that the pain was owing to a morbid growth in the globe of the eye. The sclerotic at the upper part was very much attenuated—the opake appearance in the pupil had assumed a dirty red colour, and seemed the apex of a conical-shaped body situated deep in the eye. The former treatment, with moderate ptyalism, was ineffectually adopted, and on the 19th April, Mr. Wilson removed the contents of the orbit.

"A section of the eye-ball discovered a black pultaceous tumour occupying more than one half the interior of the globe, in the situation of the vitreous humour, of which last named part, no trace could be discovered. There were two cavities or cells filled with a brownish red fluid, one situated at the side of the tumour, the other anterior to it and behind the lens. No trace of the vitreous cells could be discovered. The tunica choroides was entire, and could easily be drawn up from the sclerotica, except at one point towards its superior and internal part, where it ceased to be distinguishable from the general mass of the tumour. The sclerotica was here reduced to an extreme degree of tenuity and had a split appearance. The retina was quite detached from the choroid by the interposition of the disease, and laid folded across the globe, forming a kind of septum between the black mass and the larger of the two cavities containing the brownish red fluid. The lens was opaque and of a yellow hue, the capsule thickened, but partially transparent; a fold of retina covered the posterior capsule or that fold of the hyaloid which lines the vitreous fossula for the lodgment of the lens. The ligamentum ciliare was distinct, and some ragged portions of membrane at the margin of the lens and posterior to the iris, which was perfect, shewed a remnant of the ciliary processes. The optic nerve, where it had been divided at the time of the operation, appeared to be sound." 9.

The patient recovered from the effects of the operation, and returned home in a month, apparently well. But in August following he came back, with three or four tumours on the face, about the size of a leaden shot, perfectly black in colour, but unattended with pain. He had difficulty of breathing, stitches in his side, and a short cough. He had evidently wasted in flesh, and his pulse was quick and sharp. A tumour similar to the others was discovered on the back, between the scapulæ, and in a few days more one or two appeared on the scalp. He was now sent to the Manchester Infirmary under Dr. Home, where he remained a fortnight, and then requested his discharge. On the 2d October he came under our author's care, and the following was his condition at this time.

"The general aspect of the patient indicates a deficient supply of nutriment, or an imperfect appropriation of it to the purposes of the system. The surface is pale and exsanguineous, and there is a considerable degree of muscular emaciation with œdema of the legs. But the most striking feature of the case is an exceedingly protuberant abdomen, apparently from enlargement of one of its viscera, and this probably the liver. The tumour seems to occupy a great part of the abdominal cavity, reaching from the right to the left side and down to within an inch and a half of the pubes, where its margin can be distinctly traced. It has an irregular, tuberculated feel, is firm and unyielding over the principal extent of its surface; but the prominences of the supposed tubera possess a slight degree of elasticity. Pressure occasions little suffering; yet the patient complains of transient pains affecting both the chest and belly. He states, that, a few weeks back, he experienced a sharp pain in the left hypochondrium, darting up to the shoulder, which subsided spontaneously in the course of two or three days. He complains of a sense of weight and distention, and, indeed, his chief distress appears to arise from the mechanical influence of the swelling. The breathing is a little restrained, but there is no actual dyspnœa, and the cough which is present scarcely claims the notice of the patient. Posture somewhat affects him; he expresses himself to be easier, and his respiration freer, leaning forward at a slight angle, than when erect or in a reclining or horizontal position, though he can assume the latter without much inconvenience. The expectoration is inconsiderable, and what is brought up looks like mucus unchanged. There is less active derangement of the alimentary functions than we should have predicted, reasoning from the rapidity and extent of the hepatic disorganization. The appetite is mostly unimpaired, and the ingesta produce no disturbance to the stomach. A diarrhœa, however, harrasses the patient, but it is unaccompanied by its usual associates; and the dejections, though described as variable, are, at present, sufficiently bilious and by no means ill digested. The secretion of urine is remarkably scanty, and this fluid presents a very peculiar appearance. When emitted, it has an uniform modena red or purple colour; but by allowing it to stand some hours, a chocolate-coloured precipitate forms, leaving the supernatant fluid of a deep amber hue. Neither heat nor the nitric acid produces any visible change upon it.

"Little constitutional irritation prevails. The pulse is, indeed, somewhat accelerated, and the patient complains of transitory febrile paroxysms, which return without any assignable cause, at indefinite periods, and terminate by sweating. The tongue is clean, perhaps morbidly so, and there is some thirst.

"The face and scalp display several perfectly developed melanose tubercles, and one, on the lower lid of the extirpated eye, appears to be on the verge of ulceration, if the simple breach of its cuticular and only envelope would constitute such a state. The bottom of the orbit is free from any visible melanose deposition.* In every other situation, excepting two or three points on the trunk, the texture of the cutis has escaped the direct invasion of the disease; but the subcutaneous tissue, over the whole chest and abdomen, is evidently loaded with the morbid production which characterises melanosis; giving rise, where the cysts encroach on the skin, to faint blue elevations, more or less distinct, and of various sizes; none, however, exceeding the fourth of an inch in diameter." 14.

From this period till the 29th of the same month, the system had been gradually declining—the constitutional phenomena putting on the character of regular hectic, with colliquative perspirations, and pulse constantly above 100. The cutaneous cysts appeared to be stationary, but the sub-cutaneous were on the increase. On the 3d of November, he started suddenly from his bed in the night, being menaced with suffocation, and soon afterwards expired. The following extract is long; but we cannot possibly convey in words of our own so accurate an account of the morbid appearances in this remarkable case, as is contained in the description of our author.

"*Autopsia Cadaveris.* The body was examined nine hours after death. By making an incision from the upper part of the sternum to the symphysis pubis, and throwing back the skin, on each side, we displayed a considerable extent of the cellular and adipose textures, beautifully granulated with melanose matter; and this, at various intervals, was disposed in masses, up to the size of a small pea, all of which were inclosed in a delicate transparent cyst. Many of the largest were partially embedded in the cutis; but were held there by such slender adhesions, that the point of the knife easily dislodged them, leaving the cutaneous tissue attenuated, but otherwise unchanged. It is singular that the cellular medium of the subjacent muscles exhibited scarcely a vestige of this deposit. The remainder of the integuments was now divided, in order to expose the cavity of the abdomen. After clearing away about two quarts of bloody serum and some coagula,

"* I wish to be understood as applying this word (which is employed for the convenience of description) only to express a certain state, without any reference to the mode in which it is effected."

which had gravitated to the pelvis, we directed our observation to the liver. This viscus, completely altered in figure, was augmented to at least four times its natural magnitude; extending, laterally, to the utmost limits of the cavity, and effectually concealing the other viscera, except in the hypogastrium, where a few turns of the ilium, covered by omentum, protruded. In some places, its surface was merely undulated; in others, extremely uneven, in consequence of a number of dark blue circumscribed projections, of which the most voluminous would measure four or five inches over the summit to the base. Many of these appeared to be made up of a congregation of smaller tumours; yet, others were independent, and had probably been so from their origin. The intermediate spaces shewed little of the natural hepatic texture; its colour was converted into a faintly reddish brown, speckled with black; and in the sulci or hollows, caused by the neighbouring prominences, as well as on the surface of the more capacious tubera, the peritoneum was somewhat opaque, and, here and there, rather more vascular than usual, though in no conspicuous degree. The liver was remarkably tender and lacerable, particularly where the disorganization was most complete, so that we could not, without the utmost care, avoid breaking down its structure, in the attempt to separate and remove it. The cause of my suspicions as to abscess, now became explained; for directly opposed to that point of the umbilicus, which was the seat of my observations, we found a large tumour, projecting considerably beyond the rest, with contents sufficiently softened to convey to the finger a sense of fluctuation. It adhered slightly to the adjacent surfaces; but it is remarkable that no other morbid adhesions were discernible throughout the peritoneal cavity. On making a section of the liver, which included one of the principal tubera, several ounces of a homogeneous dark-coloured matter flowed out, bearing a near resemblance, both in look and consistence, to deep chocolate paint. We found that this product had been generated, or at least contained, in slender membranous cysts, of from one to three inches in diameter, with which the substance of the organ was beset, and that the degree of fluidity, which was most elaborated in the centre, seemed to hold a nearly direct relation to the compass of those cysts. There was besides a great number of smaller tumours, consisting of a similar but more compact material; and these, too separate or coalescent, were for the most part, bounded by a well defined cyst. In some situations, however, there was a *partial* transmutation of the parenchyma of the liver, within a circumscribed space, which appeared to be determined by the simultaneous formation of a membranous boundary, as will be seen by referring to Plate 4. In others, again, the black matter was disposed interstitially, and in a diffused manner, so as to be intermingled and confounded with the surrounding substance; and, indeed, the whole remaining hepatic structure, which was cognizable as such, betrayed a strong tincture of melanose disposition, presenting a light brown or clay-coloured ground, interspersed with innumerable dark blackish points.

“ The gall-bladder was almost buried in the disease; yet it contained its ordinary quota of fluid, in no way distinguishable from healthy bile, and its coats appeared free from any morbid deposition.

"We found the peritoneum lining the walls of the abdomen pretty generally marked with the disease; immediately, in the form of minute dots; and from poximity with the subjacent cellular tissue, in which case, the membrane was thinned, and formed but a partial envelope to small rounded tubercles, similar to what were observed in the skin. The peritoneum was elsewhere comparatively exempt from melanosis; although the reticular tissue, which united it to the subjacent structures, and connected its duplicatures, was universally pervaded by it. Along the sides of the spine in the course of the sympathetic ganglia; upon the concave surface of the diaphragm; in the adipose texture embedding the kidneys and their vessels; between the layers of the mesentery and mesocolon; and in the omentum, there was an abundant distribution of black matter, assuming various shapes, but principally, granular and ovoid.

"The pancreas, spleen, and kidneys were thickly studded with melanose bodies, some of which, especially in the pancreas, had attained the size of a Spanish nut; and we observed several patches of the same deposition beneath the serous tunic of the stomach and intestines. The former viscera were slightly increased in bulk, but preserved their respective organic characters; excepting the kidneys which were paler than usual, and in which, more particularly near the centre, the cortical and tubular divisions merged in one apparently similar structure, and this deviating from the healthy cortical only in having a weaker cohesion. At one extremity of the gland, the tubular substance looked natural; and this was the only part where melanosis had not intruded itself.

"We could distinguish no traces of the disease in the nervous textures submitted to examination; viz. the sympathetic and semilunar ganglia, and the trunks of the anterior crural and great sciatic nerves.

"The vessels, too, were free from the taint of melanosis; of which, were inspected the aorta and vena cava with their principal divisions.

"What was the nature of the lesion, which had given rise to the extravasation of blood before alluded to, we could not ascertain; for the circumstances, under which the inspection was conducted, precluded the adoption of any minute steps to determine this and some other desirable points.

"The thorax was next examined. On raising the sternum, of which the posterior surface was superficially spotted, we met with a considerable quantity of melanose tubercles lying in the cellular texture of the anterior mediastinum; but they abounded most on the exterior of the pleura costalis. On the inner surface of this membrane, as well as the pericardium, the deposit had assumed a different arrangement, being striated and so decidedly interstitial, as to form no perceptible elevation or irregularity when the finger was passed over it. The pleura investing the lungs was affected in another manner; a great number of circular or oval flattened tubercles, included in a fine transparent cyst, and attached by a slender hair-like pedicle to the subjacent membrane, was seen lying upon these organs, being congregated in some places, so as to resemble clusters of dried currants, and in others, apart and insulated, in which the filamentous attachment was especially observable. This portion of the pleura was likewise dotted interstitially, and was a little raised by the melanose bodies situated beneath it. The pulmonary substance was crepitous and had undergone no other change than in being the seat of a lightly scattered deposition, which imparted to it a carbonaceous appearance; and, here and there, though not in large quantity, the matter of melanosis was distributed in an encysted form. In no part of the chest were there any unnatural adhesions; nor did the cavities contain more than perhaps three ounces of a limpid serum. The trachea and its bifurcations presented no traces of the disease.

"Almost the whole surface of the heart was covered by melanose spots; some of which were raised, some implicating purely the texture of its investing membrane, and others again were evidently subjacent to that membrane; but none were pendent, as on the pleura pulmonalis. Further, this viscus appeared to have retained its original organic properties, only that its fibres were, here and there, separated by the interposition of a small quantity of melanose matter, and this, too, confined chiefly to within the eighth of an inch of its external surface. The membrane lining both the auricles and ventricles was perfectly healthy, and the large vessels had altogether escaped contamination."—

It is to be regretted that the brain was not permitted to be examined. On closely inspecting the product of the melanosis, it was found to possess an appearance very similar to the contents of a decaying lycoperdon (puff-ball) rendered cohesive by a small portion of liquid. It had a deep brown chocolate colour—was slightly fibrous in texture, and when agitated in water or spirit, deposited, when standing, a pulverulent sediment, the water retaining a deep tinge, but the spirit being scarcely coloured. This substance, in its most compact form, shewed a tenacity equal to that of brain. The softening was most decided in the centre of the largest tubera, and in these, the inner circumference of the cysts was fringed with flocculi of the melanose material, which were connected with it by means of a very fine cellular tissue; and this last formed the bond of union between the cyst and its contents, under all circumstances. The cysts did not present the slightest trace of vascularity, nor was there any visible turgescence in the vessels of the enclosing textures. M. Barreul, a French chemist, thinks the substance of melanose tumours is a deposition from the colouring matter of the blood and of fibrine, each under a particular modification, and forming three different fatty substances. Dr. Henry, of Manchester, analyzed a portion of the softened matter, after it had been kept sometime in spirit, and concluded, from his experiments, that the black matter is a peculiar secretion, analogous in some properties, to the colouring matter of the blood.

Our author has met with no instance on record where melanosis prevailed so universally, and proceeded with such celerity, as in the case just related. It appears to comprise nearly all the features which this rare and singular malady has been observed to assume in the different textures of the body—and exhibits, to a certain extent, the various modes by which it disorganizes or displaces those textures. It would seem that no tissue is free from the invasion of melanosis; but it attacks some in preference to others, as is exemplified in the foregoing narrative. In its progress, however, it involves, like cancer, the adjacent textures, indiscriminately destroying every thing that might oppose a barrier to its ravages. We shall not enter into any of the speculations which have been formed respecting the nature, or the causes of this dreadful disease, since we do not see that pathologists have yet been able to throw any light upon the mysterious process by which it works its fatal way in the human fabric. Neither need we say any thing of the treatment, since we are totally ignorant of any medicine capable of arresting, or even of retarding, its progress.

We need only repeat our high admiration of the plates, and assure the purchasers of this little volume that they will find in them an admirable delineation of melanosis in all the different structures of the body, and in different periods of its progress. We think the profession is greatly indebted to Mr. Fawdington for the pains which he has thus taken to elucidate an obscure, rare, and fatal malady.

